# Fetuin-A is also an adipokine

**DOI:** 10.1186/s12944-019-1021-8

**Published:** 2019-03-27

**Authors:** Ishwarlal Jialal, Roma Pahwa

**Affiliations:** 1 California North-state University College of Medicine, 9700 West Taron Road, Elk Grove, CA 95757 USA; 2 National Cancer Institute, NIH, Bethesda, MD USA

## Abstract

Fetuin-A (FetA), which impairs insulin action is considered classically as a hepatokine. In patients with Metabolic Syndrome without the confounding of diabetes or cardiovascular diseases, we showed significant increases in both circulating and subcutaneous adipose tissue secreted Fet-A. Furthermore we showed in mice models increase mRNA and protein following a high fat diet and in a model of metabolic syndrome. This work was recently confirmed by another group of investigators. Hence we propose that Fet-A be considered also as an adipokine.

Fetuin-A (Fet-A) also known as alpha-2-Heremans-Schmid glycoprotein is a 64 kDa glycoprotein that is classically considered as a hepatokine which impairs insulin action by inhibiting the auto-phosphorylation of insulin receptor tyrosine kinase [1, 2]. Fet-A levels are elevated in obesity, type2 diabetes mellitus and fatty liver disease [1, 2]. Fet-A promotes both insulin resistance and inflammation. Previously in patients with Metabolic Syndrome (MetS) without the confounding of type 2 diabetes or cardiovascular disease we have documented increased circulating and subcutaneous adipose tissue (SAT) secretion of Fetuin -A (Fet-A) which is classically considered as an hepatokine [3, 4]. The increase in SAT secreted Fet-A correlated with both plasma triglycerides and Homeostatic Model Assessment of Insulin Resistance. Because of a lack of sufficient human SAT biopsy sample, we investigated the expression (mRNA) and protein abundance of Fet-A in mice [3]. Compared to chow feeding, a high fat diet induced both mRNA and protein of Fet-A significantly in these mice. We also confirmed increase mRNA and protein for Fet-A in a mouse model of MetS, the ob/ob mice [3]. Furthermore, using the 3 T3-L1 adipocytes we showed that following differentiation over 13 days abundant expression (mRNA) of Fet-A. Hence, we concluded from these studies that Fet-A be defined as both an Adipokine and Hepatokine. Recently Perez-Stolo et al. [5] using Sprague-Dawley rats confirmed our data of increase expression and protein in AT, with more pronounced expression in visceral fat depots. They also showed that AT Fet-A could be modulated by diet and exercise and confirmed increase expression and protein in human AT from 4 donors.

Khadir et al. recently reported an increase in Fet-A in SAT of obese diabetic patients but not in circulating levels [6]. Since these patients were diabetic on multiple medications this could be a possible explanation for their failure to see increased plasma levels as they point out. This is a puzzling observation suggesting that SAT is the predominant source for functional Fet-A secretion and not the liver although we are not provided with correlations between SAT Fet-A and relevant biomarkers e.g. insulin, HbA1C etc. although SAT Fet-A was significantly increased and not plasma Fet-A. These correlations would have been most instructive. They also show that physical exercise decreased both plasma and SAT Fet-A levels in obese diabetic patients. Without providing data they state that Fet-A transcripts were not detected in SAT. However, they identified a band/transcript in 3 T3-L1 adipocytes. It is unclear, unlike most other groups, why they did not allow the adipocytes to differentiate for up to 14 days but only 8 days [3, 5]. However they should be commended in emphasizing the importance of the culture media as substrate for Fet-A especially fetal bovine serum. Finally, they present us with a schema suggesting that AT is a mere reservoir.

Given our concerns and the cogent data from 2 independent laboratories [3, 5] we humbly submit that their proposed model while based on their study is premature and posit that Fet-A be considered also as an adipokine unless refuted by other investigators. Gene Card further underscores our posit and reports transcripts in both human subcutaneous and visceral adipocytes [7].

However we should credit Khadir et al. [6] for the incremental advance for elegantly demonstrating increased adipose tissue Fet-A using confocal immunofluorescence and its decrease with exercise in obese-diabetic Kuwaiti patients.

## Abbreviations

AT: Adipose tissue
Fet-A: Fetuin-A
MetS: Metabolic Syndrome
mRNA: Messenger RNA
